# Effects of selenium supplementation on Polycystic Ovarian Syndrome: a systematic review and meta-analysis on randomized clinical trials

**DOI:** 10.1186/s12902-023-01286-6

**Published:** 2023-02-06

**Authors:** Junde Zhao, Lingfen Dong, Zhiheng Lin, Xiaohui Sui, Ying Wang, Lijuan Li, Tingting Liu, Jinxing Liu

**Affiliations:** 1 Shandong University of Traditional Chinese Medicine, 16369 Jingshi Road, Lixia District, Jinan, 250011 Shandong China; 2 Shandong University of Traditional Chinese Medicine Affiliated Hospital, Jinan, China; 3 Changchun University of Science and Technology, Jilin, 130022 Changchun China

**Keywords:** Selenium, Polycystic ovary syndrome, Meta-analysis

## Abstract

**Background:**

This study provides a systematic review and meta-analysis of randomized controlled trials, which examined the effect of the selenium supplementation on polycystic ovary syndrome (PCOS).

**Methods:**

Confirmed studies related to selenium supplementation and PCOS were searched from the databases of EMBASE, PubMed and Web of Science. Data were reported as weighted mean difference (WMD) or standard mean difference (SMD) and associated 95% confidence intervals (CIs). Analysis was performed with Stata version 12.0.

**Results:**

A total of 389 cases (selenium group *n* = 195, control group *n* = 194) were included in this studies. This meta-analysis showed that selenium supplementation has a positive effect on TAC, and supplementation of selenium does not significantly improve the level of BMI, Weight, LDL, HDL, Triglycerides, Total Testosterone, HOMA-IR, NO, GSH, MDA and FPG.

**Conclusion:**

Although selenium can improve TAC in PCOS patients, it has no significant effect on BMI, Total Testosterone, et al. In terms of the results of this meta-analysis, it is not recommended for patients with PCOS to use selenium as a regular trace element supplement. Based on the improving effect of selenium on TAC, supplementation of selenium may have a positive effect on improving follicle quality for some PCOS patients who have poor follicle quality caused by oxidative stress or who want to undergo IVF.

## Background

POLYCYSTIC OVARY SYNDROME (PCOS) is a common gynecological endocrine disease, which affects 5–20% women of reproductive age worldwide [1–3]. Its harm is not limited to infertility and abnormal menstruation [4–6], but also brings economic burden and long-term health risks to patients [7, 8]. Studies have shown that patients with PCOS often have insulin resistance and abnormal lipid metabolism [9–11]. In addition, patients with PCOS have oxidative stress [12–14]. Excessive oxidative stress and depletion of antioxidants may contribute to ovarian mesenchymal hyperplasia [15]. This affects the quality of oocytes in patients with PCOS, and ultimately leads to undesirable pregnancy [16, 17].

Selenium is an indispensable trace element for the human body and it has antioxidant and anti-inflammatory properties [18, 19]. Selenium operates as an integral part of selenoproteins assisting redox processes as an effective antioxidant [20]. Studies have shown that selenium supplementation has a positive effect on improving reproductive outcomes and inflammation biomarkers [21, 22]. It was proven that supplementing selenium increases the gene expression levels of certain enzymes and may improve lipid metabolism [23].

Many studies have confirmed that selenium supplementation has beneficial effects on patients with PCOS. It is necessary to accurately judge the effect of selenium on PCOS patients. Therefore, all relevant studies were selected and this meta-analysis was done to further confirm the effect of selenium on patients with PCOS.

## Methods

### Search strategy

A comprehensive literature search was carried out to identify all potentially relevant articles using the PubMed, EMBASE and Web of Science database from their inception to December 2022. All search methods were based on a systematic approach in line with the Preferred Reporting Items for Systematic review and Meta-Analysis Protocols (PRISMA-P). And after "snowball search", all relevant literatures were included.

Searches for terms ("Selenium"[Mesh] OR "Selenium"[Text Word] OR “Selenium-80” OR “Selenium 80”) AND ("Polycystic Ovary Syndrome"[Mesh] OR "Polycystic Ovary Syndrome"[Text Word] OR (Ovary Syndrome, Polycystic) OR (Syndrome, Polycystic Ovary) OR “Stein-Leventhal Syndrome” OR “Stein Leventhal Syndrome” OR (Syndrome, Stein-Leventhal) OR “Sclerocystic Ovarian Degeneration” OR (Ovarian Degeneration, Sclerocystic) OR “Sclerocystic Ovary Syndrome” OR “Polycystic Ovarian Syndrome” OR (Ovarian Syndrome, Polycystic) OR “Polycystic Ovary Syndrome 1” OR “Sclerocystic Ovaries” OR (Ovary, Sclerocystic) OR “Sclerocystic Ovary”) were performed. The GRADE approach was followed and uncertainty assessment per-formed for studies included in meta-analysis.

### Selection criteria

Two reviewers performed the literature search, evaluated potentially eligible studies for inclusion, and extracted the data independently. When required, disagreements were resolved by consultation with a third reviewer. Authors of the original studies were contacted for additional data, if necessary.

The main selection criteria are as follows:

- Randomized controlled clinical trials, case–control studies. Patients took selenium or selenium combined with probiotics in the selenium group. Patients took placebo in the control group.
- Study population: patients with PCOS and obesity. PCOS was diagnosed on the basis of the revised Rotterdam 2003 criteria [24]. The presence of 2 out of 3 criteria (oligo or/and anovulation, clinical or/and biochemical signs of hyperandro-genism, and polycystic ovary) was recommended as diagnostic of PCOS.
- Aged 20–40 years
- Studies that reporting weighted mean difference (WMD) or Standard mean difference (SMD) with corresponding 95% confidence intervals (95% CIs) or providing other ways to calculate or obtain these values.

### Data extraction

Two researchers extracted data from the eligible studies independently, and resolved the divergence through discussion. The information collected included author, the year of publication, age of patient, sample size, treatment method, WMD (95% CI) or SMD (95% CI), and controlled variables for matching or used in some multivariable models. The data were entered into the Review Manager software (RevMan 5.3). The quality of selected studies was evaluated by Cochrane score according to the quality standards of the Cochrane scale. The disagreement was resolved through discussion between the two reviewers. If necessary, the disagreement was resolved by consultation with the third reviewer.

### Data analysis

According to each study, 13 variables were extracted as mean ± standard deviation (SD). In this study, data were analyzed by Stata (version 12.0). P-values were two-sided, and *P* < 0.05 was considered the limit of statistical significance. In addition, the heterogeneity of these seven studies was assessed. In this meta-analysis, *I*^*2*^ was used to assess the heterogeneity between these included studies, and *I*^*2*^ ≥ 50 was set as significant heterogeneity. The random effects model was used for calculation. The WMD or SMD for continuous variables were used to explain outcomes with the 95% CI. For some data with significant heterogeneity, in-depth research was also conducted on subgroup (or regression) analysis and sensitivity analysis.

## Results

### Literature search and study characteristics

**Fig. 1:**
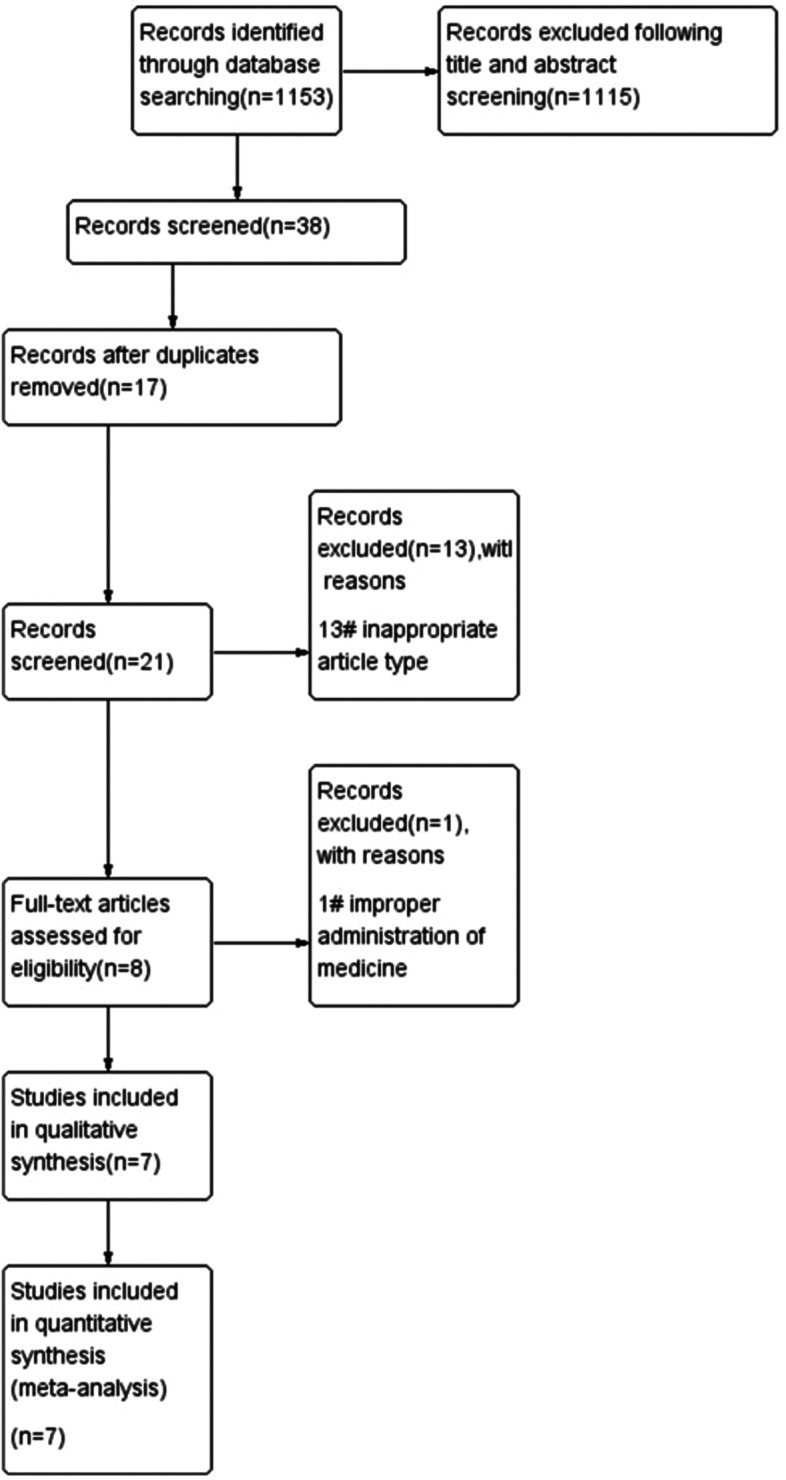
Flow Diagram of Study Selection

Figure 1 summarizes the research selection process. A total of 1098 unique references were retrieved through literature search, of which 708 were considered duplicate and irrelevant, following title and abstract screening, 17 were considered duplicate. 13 of these articles were excluded due to inappropriate article type. Of the remaining eight articles, one was excluded because of improper administration of medicine. Finally, in total, seven studies eligible for data extraction were included in the meta-analysis. Table 1 summarizes the characteristics of these studies. After "snowball search", no other literature was included.

**Table 1:** The characteristics of studies included in this meta-analysis

Study identifier	Yr	Sample		Age(yr)		Intervention	Duration of intervention	BMI at study baseline (kg/m^2^)		Weight at study baseline (kg)	
		N(s)	N(c)	Age(s)	Age(c)			BMI(s)	BMI(c)	W(s)	W(c)
Mehri Jamilian, et al. [25]	2018	30	30	26.0 ± 5.3	25.6 ± 3.8	intake 8 × 109 CFU/day probiotic plus 200 μg/day selenium supplements or placebo	12 weeks	24.6 ± 3.3	24.0 ± 3.0	63.9 ± 9.3	63.4 ± 7.7
M. Razavi, et al. [26]	2015	32	32	25.1 ± 4.5	25.4 ± 4.9	Selenium group received 200 μg daily selenium tablet as selenium yeast and Placebo group received the placebo	8 weeks	24.7 ± 3.5	25.3 ± 4.3	not available	not available
Fatemeh Mohammad Hosseinzadeh, et al. [27]	2016	26	27	29.23 ± 4.9	28.90 ± 6.1	received 200 µg selenium as a selenium-enriched yeast tablet or placebo	12 weeks	27.4 ± 4.5	28.39 ± 3.7	70.2 ± 13.8	72.6 ± 13.7
Mehri Jamilian, et al. [28]	2015	35	35	25.4 ± 5.1	25.7 ± 4.8	receive 200 µg per day selenium supplements or placebo	8 weeks	25.0 ± 3.7	25.2 ± 4.1	66.7 ± 10.0	67.1 ± 11.0
Shahrzad Zadeh Modarres, et al. [29]	2017	20	20	31.1 ± 4.7	31.4 ± 3.6	receive either 200-μg selenium as selenium yeast-free other supplements such as zinc and copper or placebo per day	8 weeks	26.5 ± 4.1	27.3 ± 2.6	69.8 ± 10.7	70.7 ± 7.1
Zahra Heidar, et al. [30]	2019	18	18	32.1 ± 4.7	32.6 ± 3.5	intake either 200 μg/day selenium as selenium yeast (Nature Made, California, USA) or placebo (Barij Essence, Kashan, Iran)	8 weeks	27.2 ± 3.1	28.6 ± 2.5	74.5 ± 8.0	76.8 ± 6.5
Batool Hossein Rashidi, et al. [31]	2019	34	32	29.4 ± 5.3	28.6 ± 5.5	receive either a daily dose of 200 μg selenium as a selenium-enriched yeast tablet or placebo	12 weeks	28.3 ± 5.2	29.5 ± 5.4	71.9 ± 14.3	74.6 ± 14.2

### Risk of bias for all studies

**Fig. 2:**
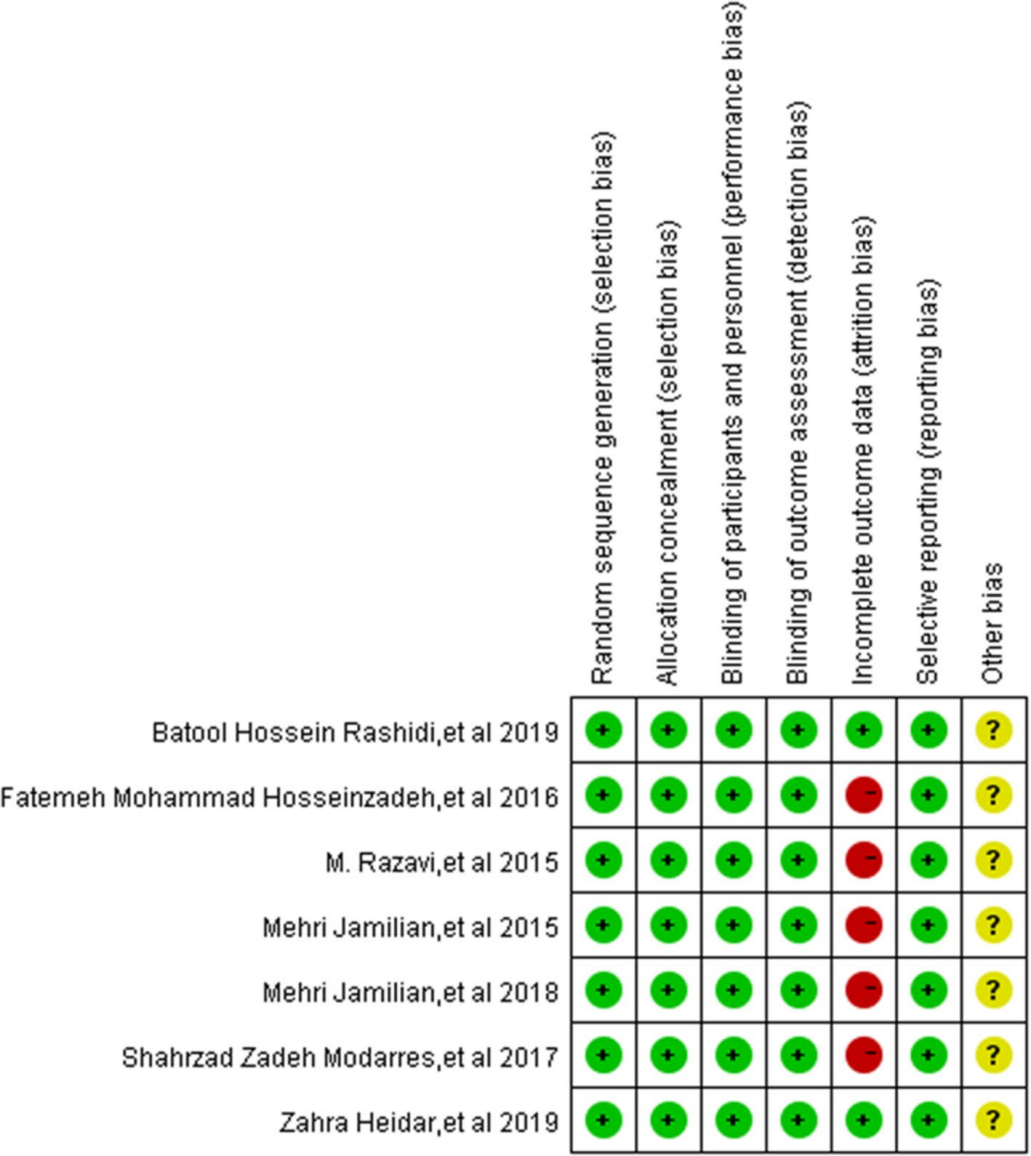
Summary of risk for each included study + ,low risk of bias;?,unknown risk of bias;-,high risk of bias

For each randomized and prospective non-randomized clinical study which was selected, the risk of bias was assessed according to the criteria described in the Cochrane Reviewers Handbook [32]. Figure 2 shows the summary of the risk of bias for each included study.

#### Effects on TAC

**Fig. 3:**
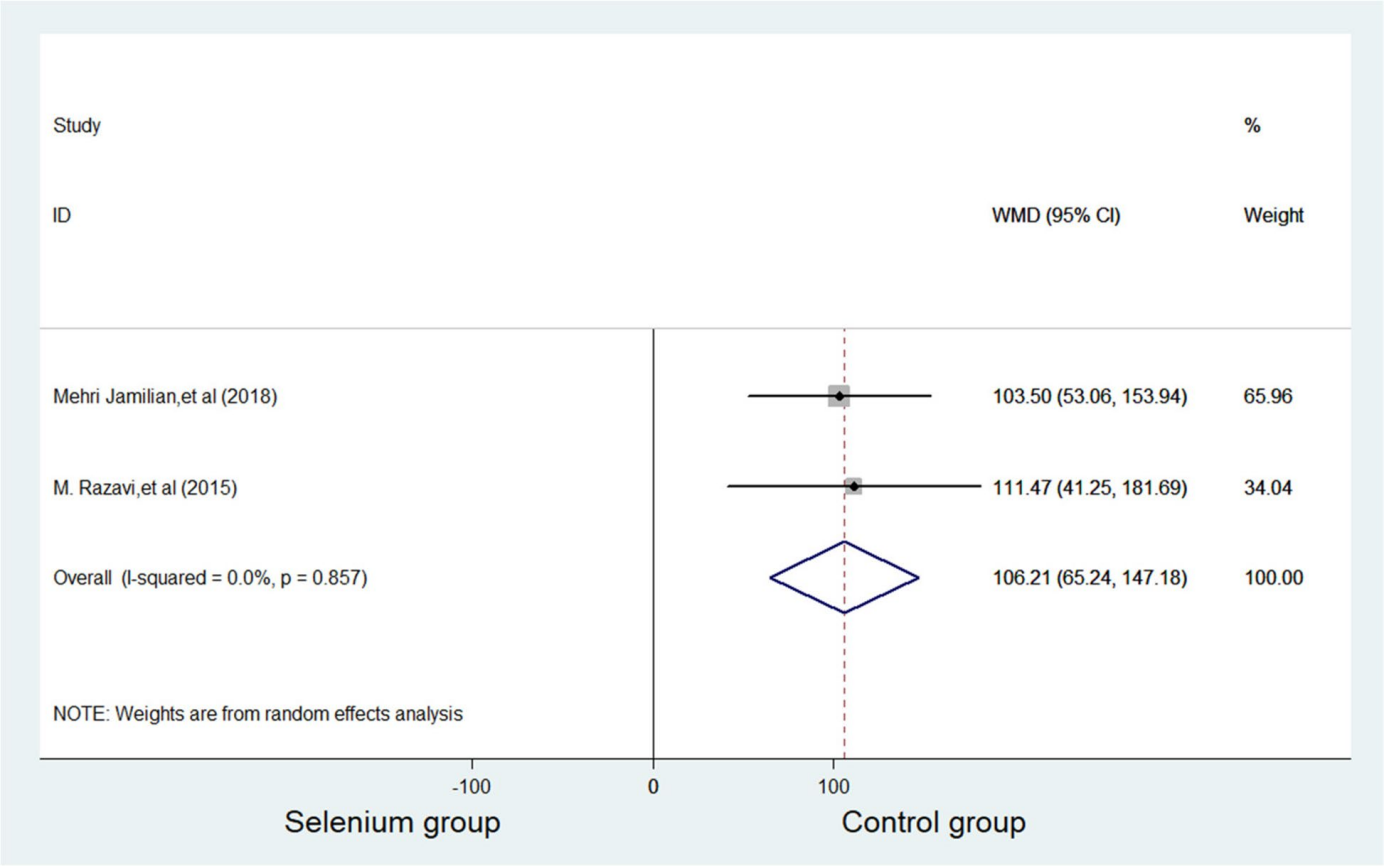
Forest plots of selenium supplementation on TAC in patients with PCOS

TAC was reported in two studies with 124 subjects, with 62 patients in the selenium group and 62 patients in control group. Compared with control group, the TAC level of selenium group increased significantly (WMD = 106.213 mmol/L, 95%CI 65.24 to 147.18, *p* ≤ 0.001). No significance was considered for heterogeneity. (*p* = 0.857, *I*^*2*^ = 0.0%) (Fig. 3).

#### Effects on SHBG

**Fig. 4:**
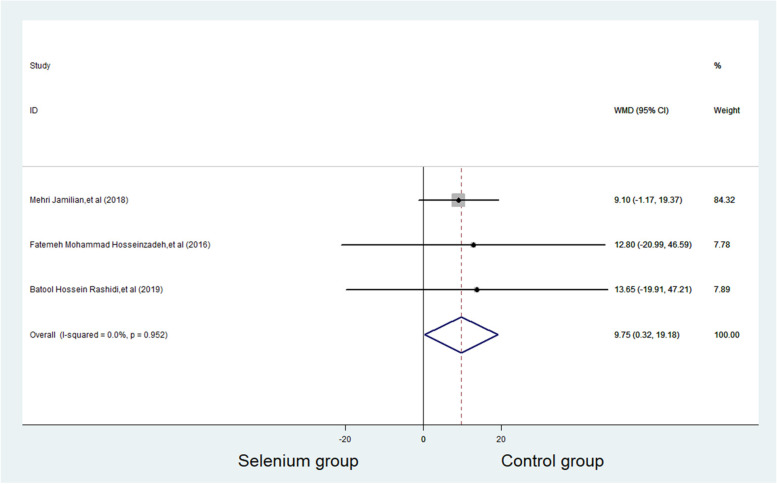
Forest plots of selenium supplementation on SHBG in patients with PCOS

**Fig. 5:**
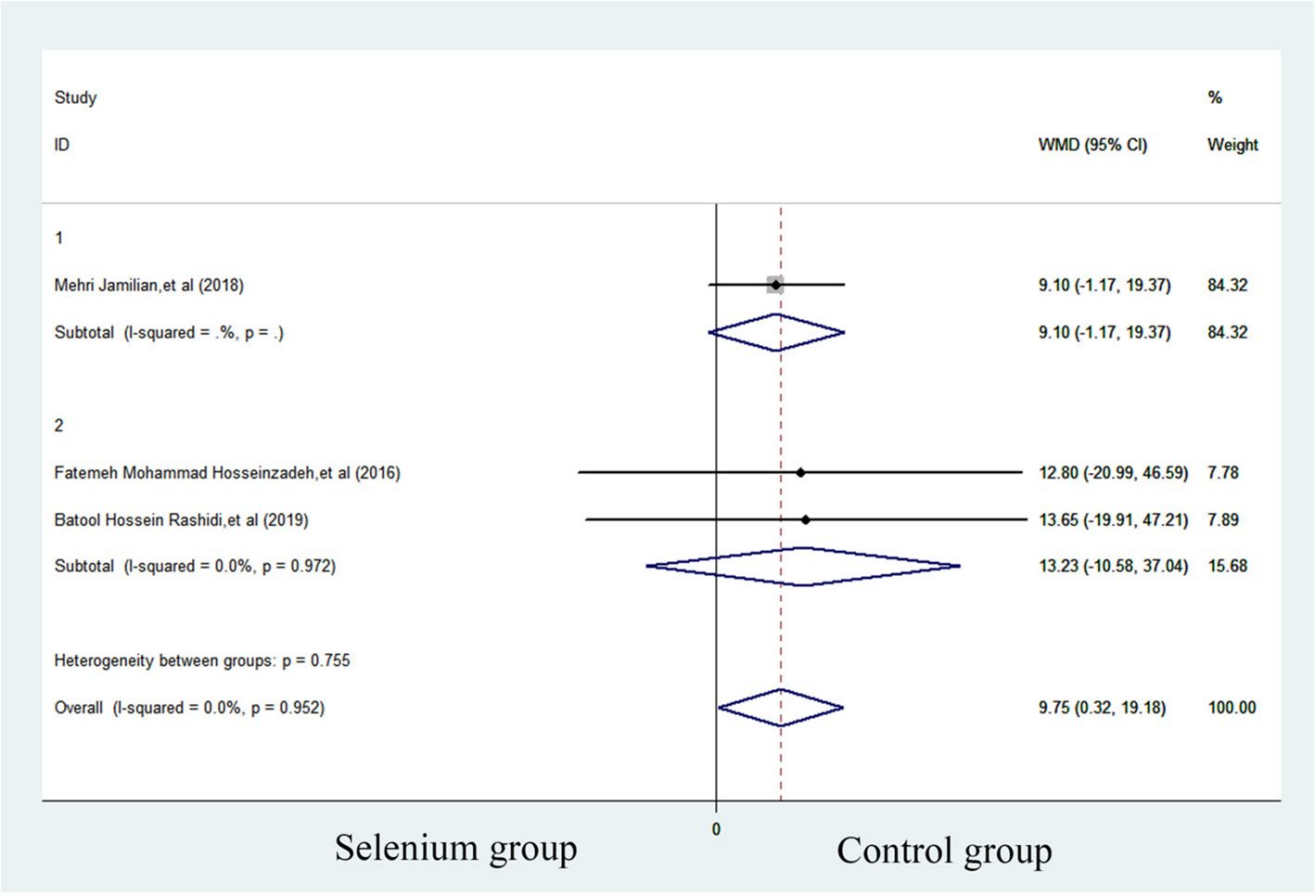
Subgroup analysis of selenium supplementation on SHBG in patients with PCOS

SHBG was reported in three studies with 179 subjects, with 90 patients in selenium group and 89 patients in control group. Compared with control group, the SHBG level of selenium group increased significantly (WMD = 9.747 nmol/L, 95%CI 0.32 to 19.18, *p* = 0.043). Heterogeneity was considered non-significant. (*p* = 0.952, *I*^*2*^ = 0.0%) (Fig. 4). In the subgroup analysis, no significant effects were observed in the subgroup of trials using selenium combined with probiotics supplements (WMD = 9.100, 95%CI -1.168 to 19.368 to 0.71) and those administrated with selenium as a single supplement (WMD = 13.228, 95% CI -10.583 to 37.039) (Fig. 5).

#### Effects on BMI

**Fig. 6:**
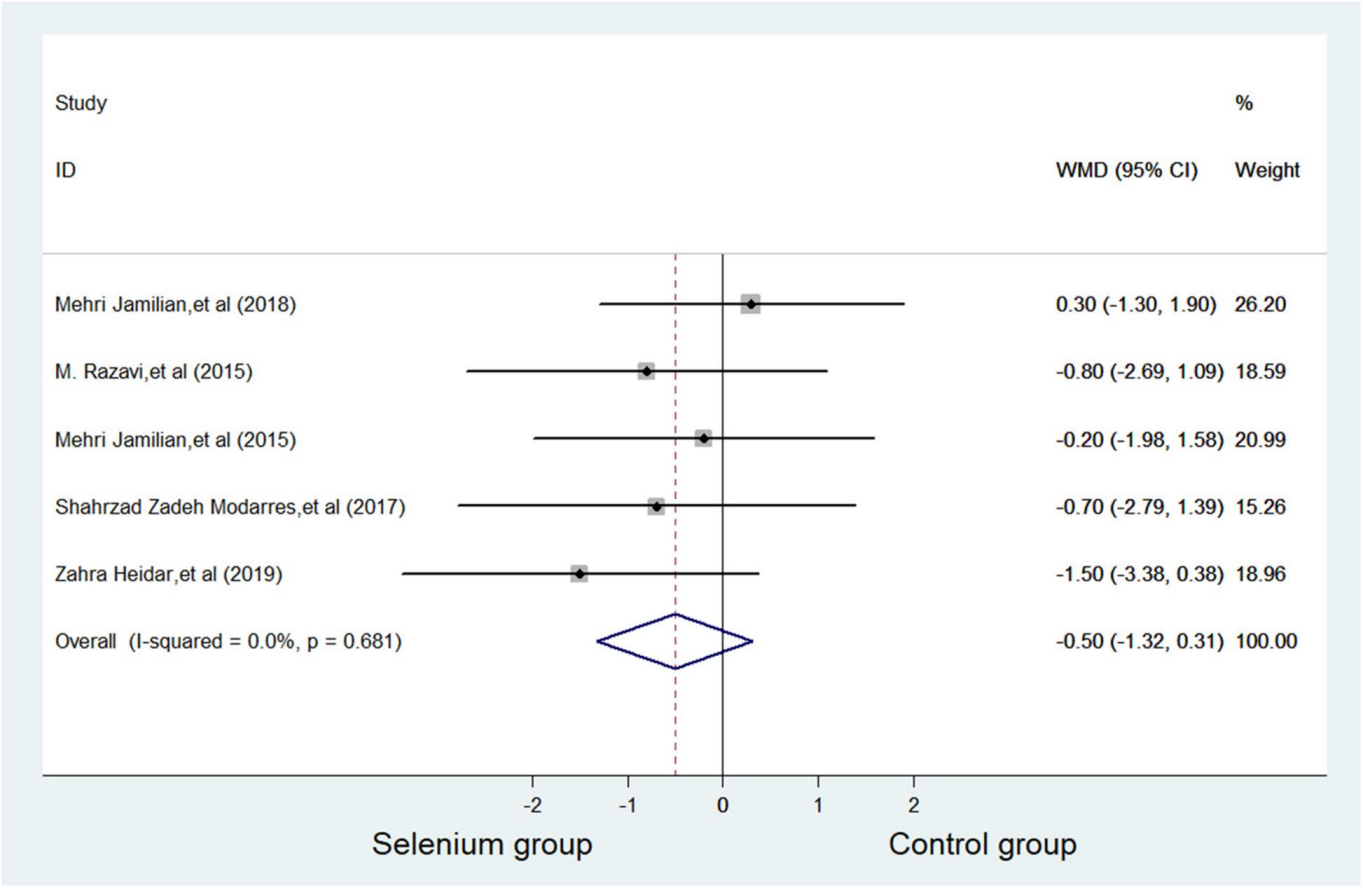
Forest plots of selenium supplementation on BMI in patients with PCOS

BMI was reported in five studies with 270 subjects, with 135 patients in selenium group and 135 patients in control group. The current meta-analysis showed that no difference in BMI was witnessed between selenium group and control group (WMD = -0.503 kg/m^2^, 95% CI -1.32 to 0.31, *p* = 0.227). No significance was considered in heterogeneity (*p* = 0.681, *I*^*2*^ = 0.0%) (Fig. 6).

### Other outcomes

**Fig. 7:**
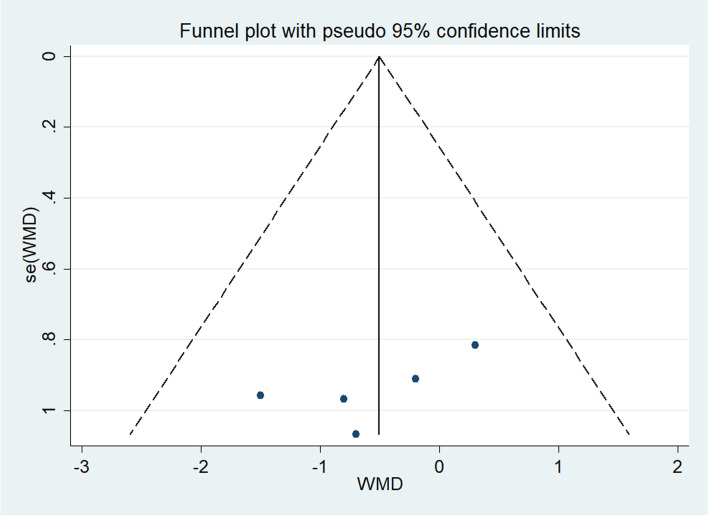
Funnel plots of publication bias. **a** Publication bias on TAC. **b** Publication bias of SHBG. **c** Publication bias of BMI

Table 2 shows a summary of the all the results of the meta-analysis, which includes the following: all outcomes: Total Testosterone, SHBG, HOMA-IR, BMI, Weight, NO, TAC, GSH, MDA, LDL, HDL, Triglycerides and FPG. Table 2 shows the results of publication bias. Figure 7 shows Begg's funnel plots estimating publication bias (Table 3).

**Table 2:** Summary of meta-analysis outcomes

Outcome	No. of studies	No. of participants	Type of meta-analysis	Effect estimate (95% CI)	*P* value	I2 (%)	Egger's test (*P* value)
Total Testosterone	3	179	WMD (fixed)	-0.14,0.11	0.767	31.8	0.991
SHBG	3	179	WMD (fixed)	0.32,19.18	0.043	0.0	0.075
HOMA-IR	3	189	WMD (random)	-1.12,0.37	0.326	53.3	0.687
BMI	5	270	WMD (fixed)	-1.32,0.31	0.227	0.0	0.182
Weight	4	206	WMD (fixed)	-3.25,1.54	0.485	0.0	0.588
NO	2	124	WMD (random)	-8.78,5.31	0.630	69.9	\
TAC	2	124	WMD (fixed)	65.24,147.18	≤ 0.001	0.0	\
GSH	2	124	WMD (random)	-39.30,91.68	0.433	64.1	\
MDA	2	124	WMD (random)	-1.16,0.43	0.373	54.3	\
LDL	2	136	SMD (fixed)	-0.49,0.18	0.359	0.0	\
HDL	2	136	SMD (fixed)	-0.48,0.19	0.405	0.0	\
Triglycerides	2	136	SMD (random)	-0.84,0.62	0.769	78.6	\
FPG	2	123	SMD (random)	-1.38,0.90	0.678	89.7	\

Values are mean ± SD; (s): selenium group (c): control group;

**Table 3:** GRADE approach

Study identifier	Type of Study	Factor of downgrade					Factor of escalation	Level of evidence
		Risk of bias	inconsistency	indirectness	imprecision	Publication bias		
Mehri Jamilian, et al. [25]	RCT	0	-1	0	0	0	None	Medium
M. Razavi, et al. [26]	RCT	-1	0	0	-1	0	None	Low
Fatemeh Mohammad Hosseinzadeh, et al. [27]	RCT	-1	0	0	0	0	None	Medium
Mehri Jamilian, et al. [28]	RCT	-1	0	0	0	0	None	Medium
Shahrzad Zadeh Modarres, et al. [29]	RCT	-1	0	0	0	0	None	Medium
Zahra Heidar, et al. [30]	RCT	-1	0	0	-1	0	None	Low
Batool Hossein Rashidi, et al. [31]	RCT	0	0	0	-1	0	None	Medium

0 Values are mean “not lower the level”, -1 Values are mean “lower one level”

## Discussion

Selenium has a significant effect of increasing TAC in patients with PCOS. The results of the study were not heterogeneous, which affirmed the antioxidant effect of selenium. There was oxidative stress in patients with polycystic ovary [9–11]. Fenkci et al. studied the TAC levels of PCOS patients and compared them with a control group matched with age, body mass index, and smoking status. They proved that TAC levels in PCOS patients were significantly reduced [33]. Oxidative stress is a state characterized by an imbalance between pro-oxidant molecules including reactive oxygen and nitrogen species, and antioxidant defenses [34]. The increased level of reactive oxygen species (ROS) deteriorates oocyte quality by inducing apoptosis [35–39]. In light of the important role that oxidative stress plays in the aetiology of oocyte function [40], it is possible that the antioxidant effects of selenium have a therapeutic role in the context of both in vitro fertilization outcomes and controlling the impact of PCSO on fertility.

SHBG can be improved with selenium, but this conclusion contradicts many studies. According to the subgroup analysis, it is clear that the overall results of the combined studies are meaningful, but two independent subgroup studies have shown that selenium supplementation alone has no effect on SHBG, and the combination of selenium and probiotics has no effect on SHBG. The reason may be that dividing the subgroups will reduce the sample size in each group. In addition, the 95% CI shows that selenium and probiotics (-1.17, 19.37) may have a more positive effect on SHBG than selenium alone (-10.58, 37.04). And it has been proven that probiotics have a significant improvement effect on SHBG [25, 41, 42]. Therefore, it is inferred that this positive result should be caused by the addition of probiotics in subgroup 1. However, it is still not clear whether the combination of probiotics and selenium has a better effect than probiotics alone.

Some limitations should be considered when the results of this meta-analysis are examined. Significant heterogeneity was founded in eligible studies on HOMA-IR, NO, GSH, MDA, Triglycerides and FPG, which has a negative impact on the meaningful results of the current meta-analysis. And because these indicators involve a small number of studies, it is impossible to accurately determine the heterogeneity source. And, the protocol of this study has not been pre-registered in PROSPERO. In addition, publication bias may slightly bias our conclusions due to the insufficient number of included studies.

**Fig. 8:**
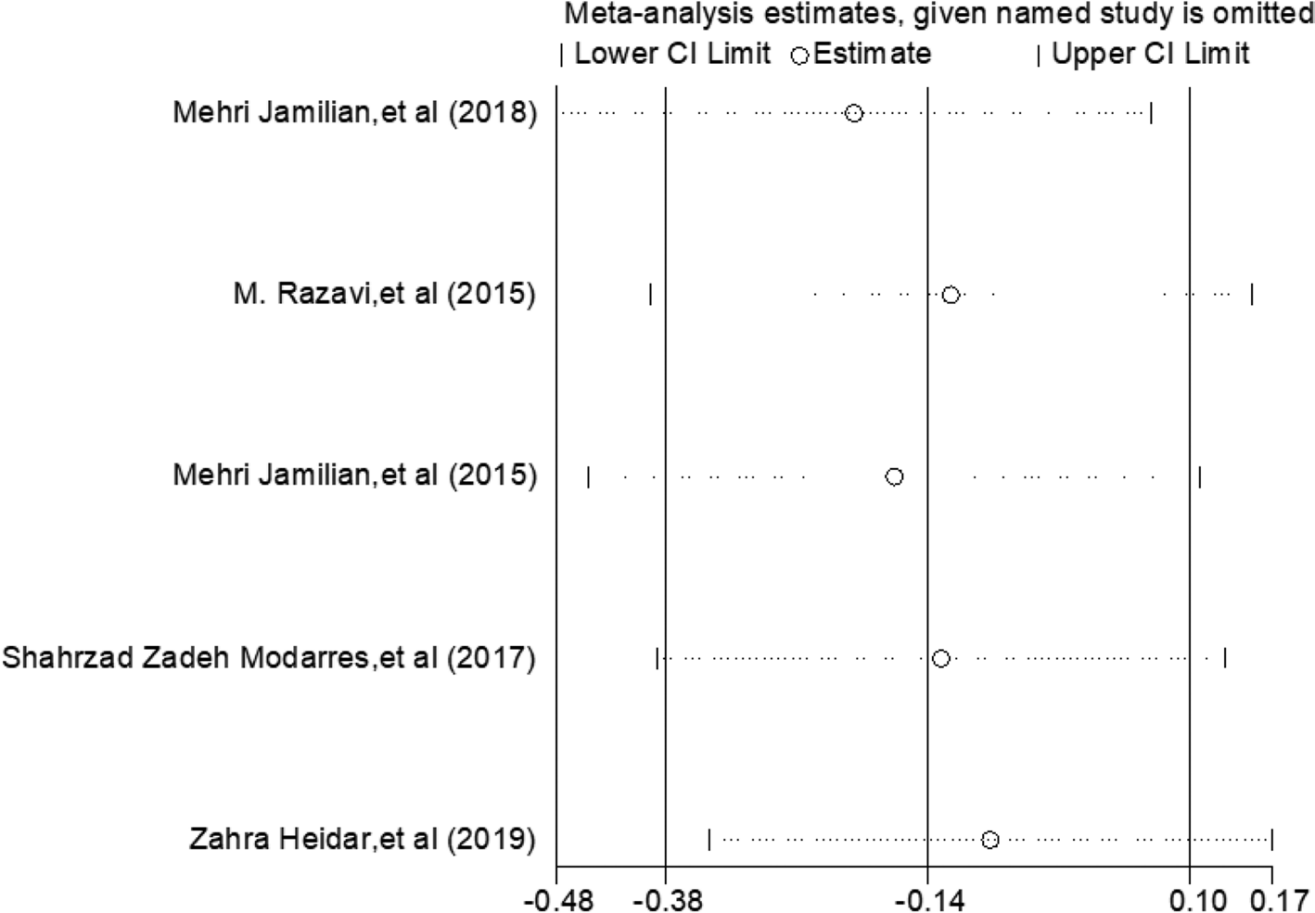
Sensitivity analysis

Despite these limitations, all existing studies on the treatment of PCOS with selenium are included, and the conclusions obtained are more valuable than a single study. And sensitivity analysis shows that the studies Mehri Jamilian, et al. [25–31] have significant sensitivity (Fig. 8) However, it is necessary to accurately determine the therapeutic effect of selenium on PCOS patients for the treatment of polycystic ovary. Therefore, more randomized controlled clinical trials of selenium in the treatment of PCOS are suggested to prove the effects of selenium on all aspects of PCOS patients.

## Conclusion

Regardless of its positive effect on TAC, this meta-analysis shows that supplementation of selenium does not significantly improve the level of BMI, Weight, LDL, HDL, Triglycerides, Total Testosterone, HOMA-IR, NO, GSH, MDA and FPG. Therefore, in terms of the results of this meta-analysis, it is not recommended for patients with PCOS to use selenium as a regular trace element supplement. As for some PCOS patients who have poor follicle quality caused by oxidative stress or who want to undergo IVF, supplementation of selenium may have a positive effect on improving follicle quality.

## Data Availability

All the data in this paper support the results of this study.

## Abbreviations

CI: Confidence interval
PRISMA: Preferred Reporting Items Systematic Reviews and Meta-Analyses
WMD: Weighted mean difference
SMD: Standard mean difference
RCT: Randomized controlled trial
PubMed: National Library of Medicine
EMBASE: Excerpta Medica Database
PCOS: Polycystic ovary syndrome
LDL: Low-density lipoprotein
SHBG: Sex hormone binding globulin
BMI: Body mass index
HDL: High-density lipoprotein
HOMA-IR: Homeostasis model of assessment-insulin resistance
FPG: Fasting plasma glucose
NO: Nitric oxide
MDA: Malondialdehyde
TAC: Total antioxidant capacity
GSH: Total glutathione
